# Small animal look-locker inversion recovery (SALLI)

**DOI:** 10.1186/1532-429X-13-S1-P24

**Published:** 2011-02-02

**Authors:** Daniel Messroghli, Sarah Nordmeyer, Martin Buehrer, Sebastian Kozerke, Thore Dietrich, Thomas Hucko, Felix Berger, Christoph Klein, Titus Kuehne

**Affiliations:** 1 German Heart Institute Berlin, Congenital Heart Defects and Pediatric Cardiology, Berlin, Germany; 2 Institute for Biomedical Engineering, ETH and University of Zurich, Zurich, Switzerland; 3 Department of Cardiology and Internal Medicine,German Heart Institute Berlin, Berlin, Germany

## Introduction and purpose

Cardiac T1 mapping allows for quantitative analysis of myocardial tissue properties. Pulse sequences for human applications are not suitable for in-vivo studies in small animals.The aim of this study was to develop a single magnetic resonance imaging (MRI) approach for comprehensive assessment of cardiac function and tissue properties in small animals with high heart rates.

## Methods

Small animal Look-Locker inversion recovery (SALLI) was implemented on a clinical 3 Tesla MRI system equipped with a 70 mm solenoid coil. SALLI combines a segmented, ECG-gated, IR-prepared Look-Locker type pulse sequence with a multimodal reconstruction framework. The pulse sequence scheme uses radial non-balanced steady-state free precession readout to minimize motion artifacts. Data acquisition can be accelerated through the use of temporal undersampling by a factor of R, where only a fraction of 1/R profiles is acquired for each heart phase while still allowing for reconstruction of fully sampled data sets. To assess T1 accuracy in-vitro, SALLI with different sequence settings was performed in 9 agarose gel phantoms. In-vivo, 10 Sprague-Dawley rats were studied to establish normal values pre- and post- injection of gadopentetate dimeglumine. One rat with surgically induced acute myocardial infarction was examined to test the potential of SALLI for the detection of acute myocardial injury.

## Results

**Figure 1:**
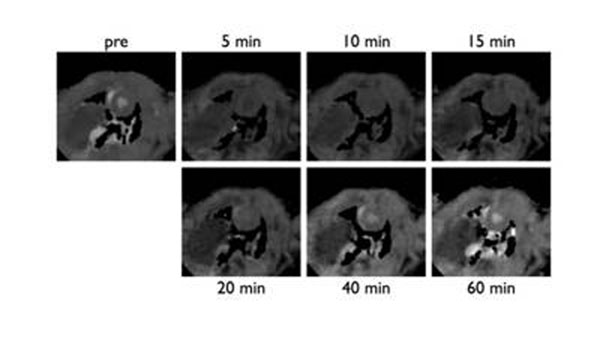
Representative set of T1 maps before (pre) and after (5 to 60 minutes) intravenous application of Gd-DTPA in a healthy male Sprague-Dawley rat. Field-of-view 65 x 65 mm, pixel size 0.60 x 0.60 mm, slice thickness 3.0 mm, TR 5.2 ms, TE 2.2 ms, FA 10°.

Phantom studies demonstrated systematic behavior of T1 measurements. In-vitro T1 error could be reduced to 1.3±7.4% using a simple linear correction algorithm. Figure 1 shows a representative set of T1 maps from one of the wild-type rats at different time points. Pre- and post-contrast T1 of left-ventricular myocardium and blood showed narrow normal ranges. In the animal with surgical ligation of the left circumflex artery, SALLI demonstrated hypokinesia (cine images), myocardial edema (pre-contrast T1 map), and myocardial necrosis (post-contrast T1 map and late gadolinium enhancement) in the area of infarction

## Conclusions

SALLI enables simultaneous generation of cardiac T1 maps, cine, and inversion recovery (IR)-prepared images at high heart rates. T1 measurements demonstrate high accuracy in-vitro and narrow normal ranges in-vivo. SALLI might allow for comprehensive qualitative and quantitative assessment of myocardial morphology and function in small animal models of myocardial injury.

